# The core genetic drivers of chronological aging in yeast are universal regulators of longevity

**DOI:** 10.15698/mic2025.10.861

**Published:** 2025-10-31

**Authors:** Erika Cruz-Bonilla, Sergio E. Campos, Soledad Funes, Cei Abreu-Goodger, Alexander DeLuna

**Affiliations:** 1 Unidad de Genómica Avanzada, Cinvestav, 36824 Irapuato, Mexico.; 2 Centro de Investigación sobre el Envejecimiento, Cinvestav, 14330 Tlalpan, Cd.Mx., Mexico.; 3 Departamento de Genética Molecular, Instituto de Fisiología Celular, Universidad Nacional Autónoma de México, 04510 Coyoacán, Cd.Mx., Mexico.; 4 Institute of Ecology and Evolution, The University of Edinburgh, EH9 3FL Edinburgh, UK.

**Keywords:** chronological lifespan, genome-wide mutant screening, functional genomics meta-analysis, genetic interactions, Saccharomyces cerevisiae

## Abstract

The chronological lifespan of *Saccharomyces cerevisiae* has significantly contributed to our understanding of aging in eukaryotic cells. However, gaining a genome-wide perspective of this trait remains challenging due to substantial discrepancies observed across genome-wide gene-deletion screens. In this study, we systematically compiled nine chronological-lifespan datasets and evaluated how shared experimental variables influenced screen variability. Furthermore, we performed a meta-analysis to compile a ranked catalog of key processes and regulators driving chronological longevity in yeast, ensuring their robustness across diverse experimental setups. These consistent chronological aging factors were enriched in genes associated with yeast replicative lifespan and orthologs implicated in aging across other model organisms. Functional analysis revealed that the downstream cellular mechanisms underlying chronological longevity in yeast align with well-established, universal hallmarks of aging. Importantly, we identified transcriptional regulators associated with these consistent genetic factors, uncovering potential global and local modulators of chronological aging. Our findings provide an integrated view of the core genetic landscape underlying aging in yeast, highlighting the value of the chronological lifespan paradigm for investigating conserved mechanisms of aging.

## Abbreviations

CFU - colony-forming units,

CLS - chronological lifespan,

RLS - replicative lifespan,

ROC - receiver operating characteristic,

TF - transcription factor.

## INTRODUCTION

Lifespan is a complex biological trait shaped by the rate of aging, which is influenced by both genetic and environmental factors 1. Model organisms have been instrumental in uncovering the intricate genetic mechanisms underlying aging and longevity. Notably, the *age-1* phosphoinositide 3-kinase that regulates the insulin/IGF-1 signaling pathway, was the first "aging gene" identified in *Caenorhabditis. *The sole mutation of this gene extends lifespan by up to 65% 2. Similarly, mutations in the insulin/IGF-1 receptor gene *daf-2* extend the lifespan of *Drosophila *3, while mice lacking the insulin receptor in adipose tissue also live longer 4. These findings underscore the evolutionary conservation of aging pathways across diverse species.

The budding yeast *Saccharomyces cerevisiae* serves as a valuable model for studying aging, providing powerful tools for genetic manipulation in a unicellular organism with a short lifespan 5. Aging is modeled in yeast cells by analyzing their replicative or chronological lifespans 6. The replicative lifespan (RLS) refers to the number of mitotic cell divisions that a mother cell can undergo, while the chronological lifespan (CLS) is defined as the number of days a non-dividing population remains viable in stationary phase. Introduced in the 1990-2000 7,8,9,10, the CLS paradigm has since facilitated the progressive elucidation of a complex regulatory network underlying aging and involving nutrient-sensing pathways, mitochondrial metabolism, reactive oxygen species signaling, autophagy, and diverse stress-response mechanisms. Key nutrient-sensing pathways such as TOR1 and RAS/PKA, when inhibited or modulated through caloric or dietary restrictions, have been shown to enhance mitochondrial efficiency and trigger adaptive hormetic responses that extend lifespan 9,11,12,13,14,15,16,17. Additionally, autophagy consistently emerges as an essential process to maintain cellular homeostasis by clearing damaged cellular components, thus delaying chronological aging 18,19. Despite significant advancements, the nature and complex interplay among these regulatory elements continues to be dissected and an integrated understanding of chronological aging modulators remains incomplete.

The CLS of yeast cell populations is conventionally measured by following changes in colony-forming units (CFU) as a function of time in stationary phase cultures 20,21,22,23. Using alternative high-throughput approaches, the entire yeast genome has been successfully surveyed for CLS genetic factors 24,25,26,27,28,29,30,31. These unbiased studies have uncovered additional pathways, including potential roles for genes involved in tRNA modification 26, *de novo* purine biosynthesis 25, the SWR1 chromatin remodeling complex 30, and the MAPK signaling cascade 31. However, validating genome-wide screens of CLS phenotypes has proven to be challenging, as false-positive hits range from 50% to over 90% when mutants are tested individually using standard CFU methods 24,25,26,30,31. Moreover, minimal overlap has been documented in the lifespan modifiers identified across different genome-wide CLS screens. For example, only nine and 22 out of over 800 long- and short-lived mutants, respectively, are shared among three genome-wide screens 32. Variations in media composition, genetic backgrounds, and phenotyping methodologies likely contribute to the limited consistency observed, particularly for long-lived mutant phenotypes.

In this study, we aimed to identify genes consistently recognized as lifespan modulators in large-scale CLS screens, along with their associated features in *S. cerevisiae* and other model organisms. Using meta-analysis of a compiled set of studies, we obtained a unified, ranked catalog of genes and functions that impact CLS regardless of conditions or experimental setups. We evaluated the distribution of genes associated with RLS phenotypes and aging orthologs in metazoans, revealing parallels with the spectrum of CLS factors. Functional analysis of the ensuing longevity rank highlights both well-established modulators of aging in eukaryotes and less explored pathways that warrant further investigation, underscoring the value of the yeast CLS paradigm in aging research.

## RESULTS

### Overview of genome-wide gene-deletion screens of yeast chronological lifespan

**Figure 1:**
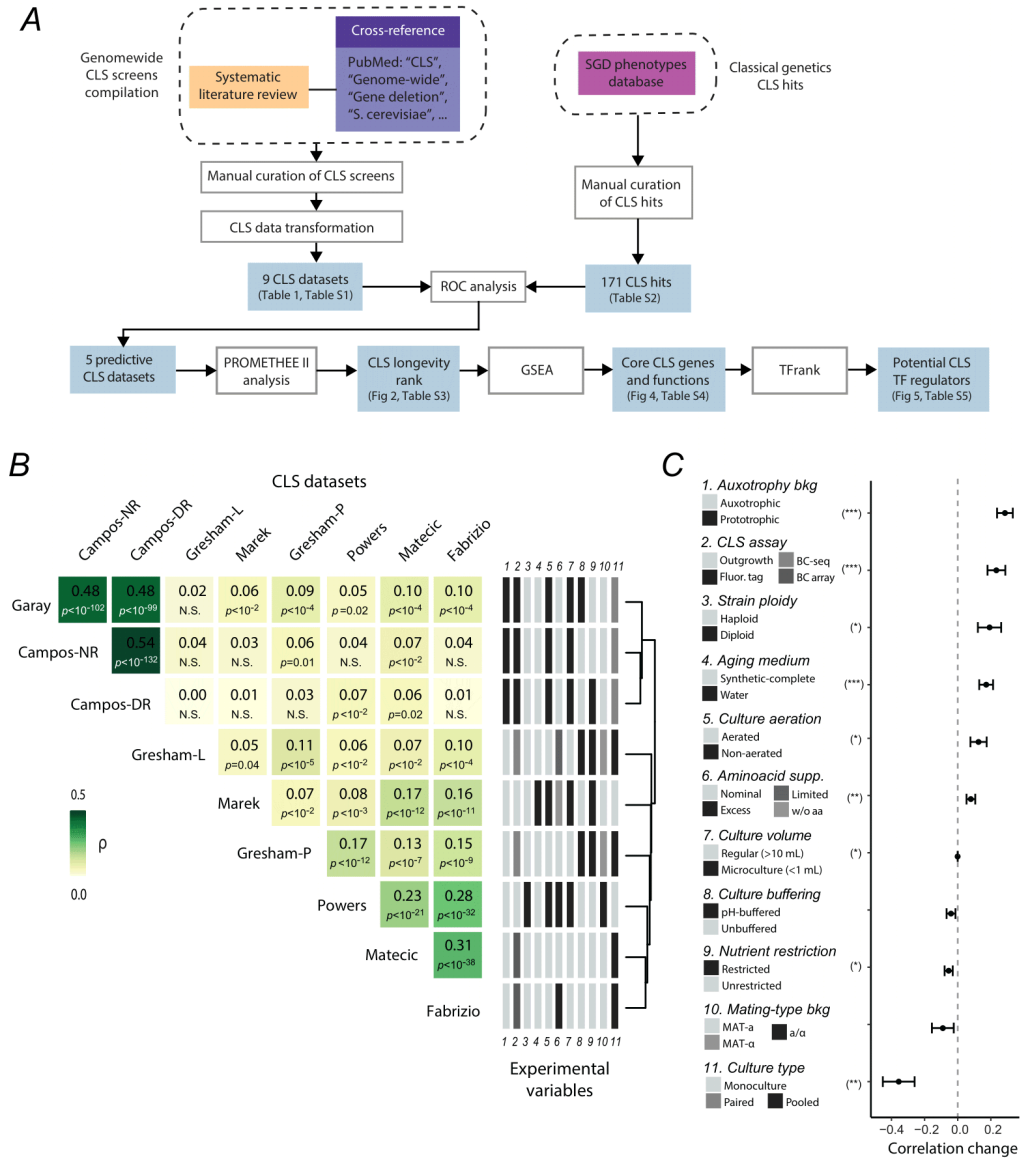
FIGURE 1: Systematic compilation and comparison of genome-wide CLS screens. **(A) **Schematic workflow summarizing the analyses performed in this study. **(B) **Modest correlation among genome-wide CLS assays. Spearman rank correlation matrix of CLS datasets; gene deletions were ranked from highest to lowest lifespan according to each assay’s measurements, and pairwise Spearman correlation coefficients and associated *p*-values were calculated. Only genes shared across all datasets (n = 1751) were included. Datasets are ordered based on hierarchical clustering of their correlation coefficients. Eleven associated experimental variables are shown to the right of the matrix. **(C)** A linear model was used to calculate the average changes in pairwise correlation based on shared experimental variables. Asterisks indicate experimental variables significantly contributing to correlation changes of at least ±0.1 (*p<0.05, **p<0.01, ***p<0.005).

We performed a targeted PubMed search to identify original research articles published up to 2023 that report genome-wide surveys of genes affecting survival of *S. cerevisiae* cells under non-dividing conditions (**Figure 1A**). The search combined specific terms related to life span regulation ("chronological lifespan", "lifespan regulation", "stationary phase survival", "nutrient starvation") with experimental approaches ("genome-wide", "gene deletion", "profiling", "system-level") and restricted results to articles mentioning "*Saccharomyces cerevisiae*" in the title or abstract. This strategy yielded a focused list of 21 primary studies, which were manually reviewed. Studies were excluded if they did not directly assess survival phenotypes (e.g., transcriptomic analyses), did not report original lifespan data of gene-deletion strains, or were conducted in other model organisms. Among the studies meeting these criteria, only one large-scale *S. cerevisiae* screen was excluded because it covered less than 70% of the non-essential genome 26.

After this systematic review of the literature, seven references were retained, which included nine genomewide mutant screens, accounting for differences in experimental conditions within the assays (e.g. media conditions) (**Table 1**). The CLS data of this final screen selection was acquired and compiled into Table S1 (see Materials and Methods) and are hereafter referred to as the CLS datasets. These originated from six different research groups, all using strains derived from the S288C yeast deletion collection. The large-scale surveys differed in several aspects, including strain ploidy, amino acid auxotrophies, culture conditions, and media composition; differences in the screening methodology were also present. These final datasets are provided in Table S1 (see Materials and Methods). Across the nine resulting datasets, CLS phenotypic data was available in at least one strain of 4779 deletion strains in total (Figure S1).

### Genome-wide screens exhibit modest overlap in the resulting CLS phenotypes

To evaluate the agreement across studies, we compared the pairwise correlation of the CLS results available in all datasets (1751 strains, 36.6% intersection). Since each study reports CLS phenotypes using different quantitative scales, we used their ranked phenotypes, from the most long-lived to the most short-lived mutant in each dataset. In agreement with previous observations based on three genome-wide screens 30, modest to no rank correlation was observed between the datasets (**Figure 1B**; ρ<=0.54). The highest pairwise similarities were observed among the ‘Garay’, ‘Campos-NR’, and ‘Campos-DR’ datasets (ρ=0.48 - 0.54, *p*<10^-99^), as expected given the same genetic background and experimental approach used in these studies. Likewise, the ‘Fabrizio’ and ‘Matecic’ datasets were highly correlated with each other (ρ=0.31*, p*<1.1x10^-39^), in agreement with the similar microarray barcode approach used. Given the high frequency of gene deletions with neutral or nearly neutral phenotypes, we also compared the overlap among cutoffs of long- and short-lived strains, estimated from their Jaccard similarity coefficients. This analysis confirmed only modest overlap in CLS-segmented phenotypes between studies (Figure S2). For instance, the Jaccard overlap index ranged between 0.04 and 0.29 for the most stringent cutoff for long-lived strains, while the corresponding indexes were between 0.1 and 0.38 for short-lived strains. Overall, higher overlap was observed in short-CLS phenotypes in comparison to long-CLS, as previously observed by Smith *et al*. 32. These findings confirm that genome-wide CLS-phenotype screens exhibit only modest overlap, even when experimental conditions differ only slightly.

### Evaluating the impact of experimental variables on CLS screen consistency

In an effort to explain screen variability, we applied a linear model to predict the correlation between pairs of datasets, incorporating whether they shared particular experimental conditions as variables. This model explained an important part of the variability in the pairwise correlations, suggesting that at least some experimental variables significantly influence the consistency between screens (*R_adj_*^2^=0.80, *p*<10^-7^). We then examined how each variable affected the average correlation between datasets when shared, compared to when it was not. Notably, the use of auxotrophic versus prototrophic strains had the strongest positive impact on correlation values, indicating that differences in auxotrophy background markedly reduce reproducibility between screens (**Figure 1C**). The CLS screening strategy itself also contributed significantly to the observed variability, while other factors such as ploidy, medium composition, and aeration had a smaller yet measurable impact. Interestingly, variables commonly assumed to strongly affect CLS phenotypes, such as pH buffering 33 and dietary restriction 12, showed minimal contribution to correlation, suggesting their interaction with gene deletions may not be as influential at the genome-wide scale. Intriguingly, the type of strain setup—whether monoculture, competitive aging, or pooled deletions—had a strong and significant negative effect on correlation, possibly reflecting confounding from unaccounted experimental factors or more complex interaction effects. Together, these findings provide an overview of the experimental sources of variation in CLS screens, offering practical insights for designing more reproducible large-scale aging studies in yeast.

### A ranked catalog of genes robustly influencing CLS in yeast 

It is reasonable to assume that there are fundamental mediators of chronological aging and longevity that impact CLS regardless of the conditions or experimental setup, though their relative impacts may vary. To identify such ‘core’ set of genetic factors consistently associated with CLS phenotypes across diverse experimental conditions, we employed the PROMETHEE outranking approach 34. This method is well suited for integrating heterogeneous datasets, as it enables the ranking of alternatives (genes) based on pairwise comparisons of their performance across multiple criteria—in this case, CLS phenotypes from different studies. The resulting preference scores (*phi-scores*) reflect how strongly each gene is supported to have higher CLS relative to others across datasets. Unlike threshold-based methods, PROMETHEE can highlight gene deletions with moderate but consistent phenotypes that might be missed in individual screens. Crucially, this approach accommodates the variability in measurement scales and the fact that different sets of mutants are included in the studies, making it especially appropriate for comparative analysis of the compiled datasets 33.

The PROMETHEE method also allows for establishment of criteria relevance via weight allocation. To establish the relative relevance of each dataset in the final ranking, we compiled a list of 171 gene deletions with CLS phenotypes that had been scored in smaller-scale studies using standard CFU 22 or live/dead staining approaches 23 (**Figure 1A**; Figure S3; Table S2). We used this curated set of CLS phenotypes to assess the predictive performance of each of the genome-wide screens and account within the final ranking for known issues of false-positive rates within datasets. Specifically, we calculated a receiver operating characteristic (ROC) curve for each CLS dataset, where dataset performance was evaluated by accurately classifying the lifespan phenotypes within the curated CLS phenotype set (Figure S3, Materials and Methods). Subsequently, we settled for five datasets based on their predictive performance, as manifested on their ROC curves; only one dataset per study was included for a more extensive set of experimental conditions. Finally, by assigning equivalent weights to each dataset as predictive performance for selected datasets was similar, we obtained a final ranked list, which included all genes with available phenotypes in at least one of the five datasets used in our PROMETHEE outranking approach. **Figure 1A** provides a schematic overview of the procedure used to generate the ranked catalog of CLS factors. We note that alternative rankings based on the different weight-allocation parameters were also analyzed, which resulted in similar rankings at the extremes of the ranks (Figure S4; Table S3).

**Figure 2:**
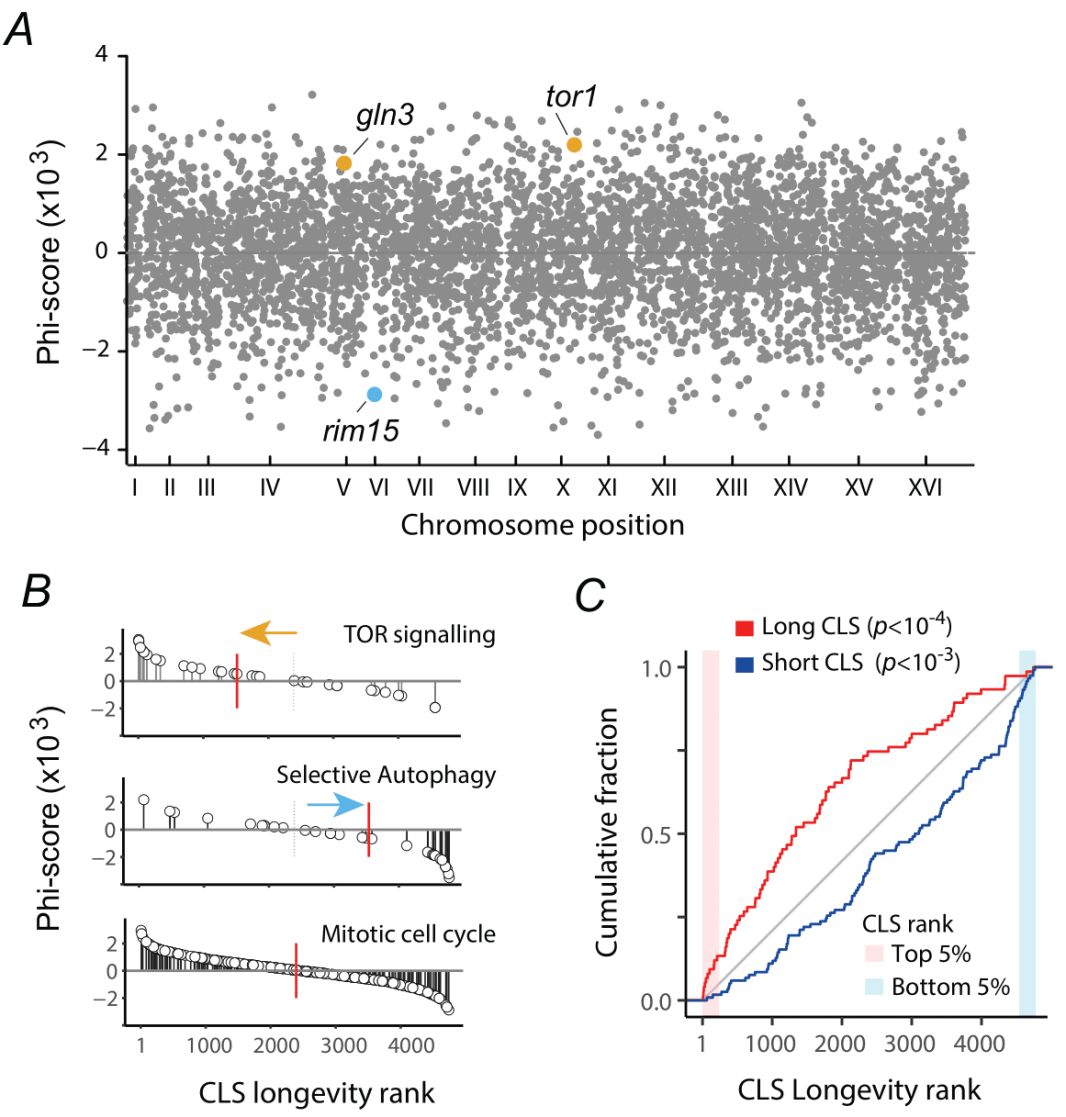
FIGURE 2: A ranked catalog of consistent CLS phenotypes for budding yeast. **(A)** *Phi-score* of genes across the yeast genome, reflecting evidence of increased (*phi*>0) or decreased (*phi*<0) longevity upon gene deletion. Some CLS modulators are highlighted: *TOR1* and *GLN3* (long-lived mutants) and *RIM15* (short-lived mutant). **(B)** Plots show the *phi-score* of genes within three example functional categories. Red lines are the median rank within each set, and colored arrows indicate deviation from these median values. **(C)** Plot shows the cumulative distributions of long-lived (red) and short-lived (blue) gene deletions from the curated CLS phenotype set (Table S2) across the CLS longevity rank, compared to the null expectation of a random distribution shown in gray (Wilcoxon rank sum test). Shaded areas indicate the top and bottom 5% of the CLS longevity rank.

Our meta-analysis approach resulted in a final ranked list of 4779 CLS phenotypes. The ensuing CLS longevity rank provides a genome-wide picture of genes influencing the chronological aging of yeast in a consistent manner, regardless of the experimental setup (**Figure 2A**; Table S3). Importantly, the assigned outranking *phi-score* of each gene reflected the weight of its phenotype relative to other genes across studies. For instance, top-ranked genes with high *phi-score*s were those with more robust evidence of increased longevity when deleted, which included *TOR1* (ranked 74, top 1.5%), *GLN3* (ranked 170, top 3.5%), and other genes involved in TOR signaling (**Figure 2A, B**). Genes within the lowest longevity ranks and negative *phi-score*, such as *RIM15 *(ranked 4742, bottom 0.8%) and several autophagy (*ATG*) genes, were more likely to be short-lived mutants. As expected for the postmitotic survival phenotypes herein analyzed, genes associated with the mitotic cell cycle showed a uniform distribution in the rank, with no evident enrichment at either extreme of short- or long-lived phenotypes (**Figure 2B**). Furthermore, we confirmed that long-lived gene deletions from the curated catalog of 171 CLS phenotypes were significantly biased toward the top of the ensuing CLS longevity rank, while short-lived mutants were more common at the bottom of the rank (**Figure 2C**). These curated phenotypes were significantly enriched at the bottom 5% (*p*<0.0015, hypergeometric test) and marginally enriched in the top 5% (p<0.0517) of the CLS longevity rank. These general features of the ensuing ranked catalog suggest that it provides a valuable resource to interrogate the functional features robustly associated to CLS genetic factors in yeast.

### CLS factors are usually pleiotropic

**Figure 3:**
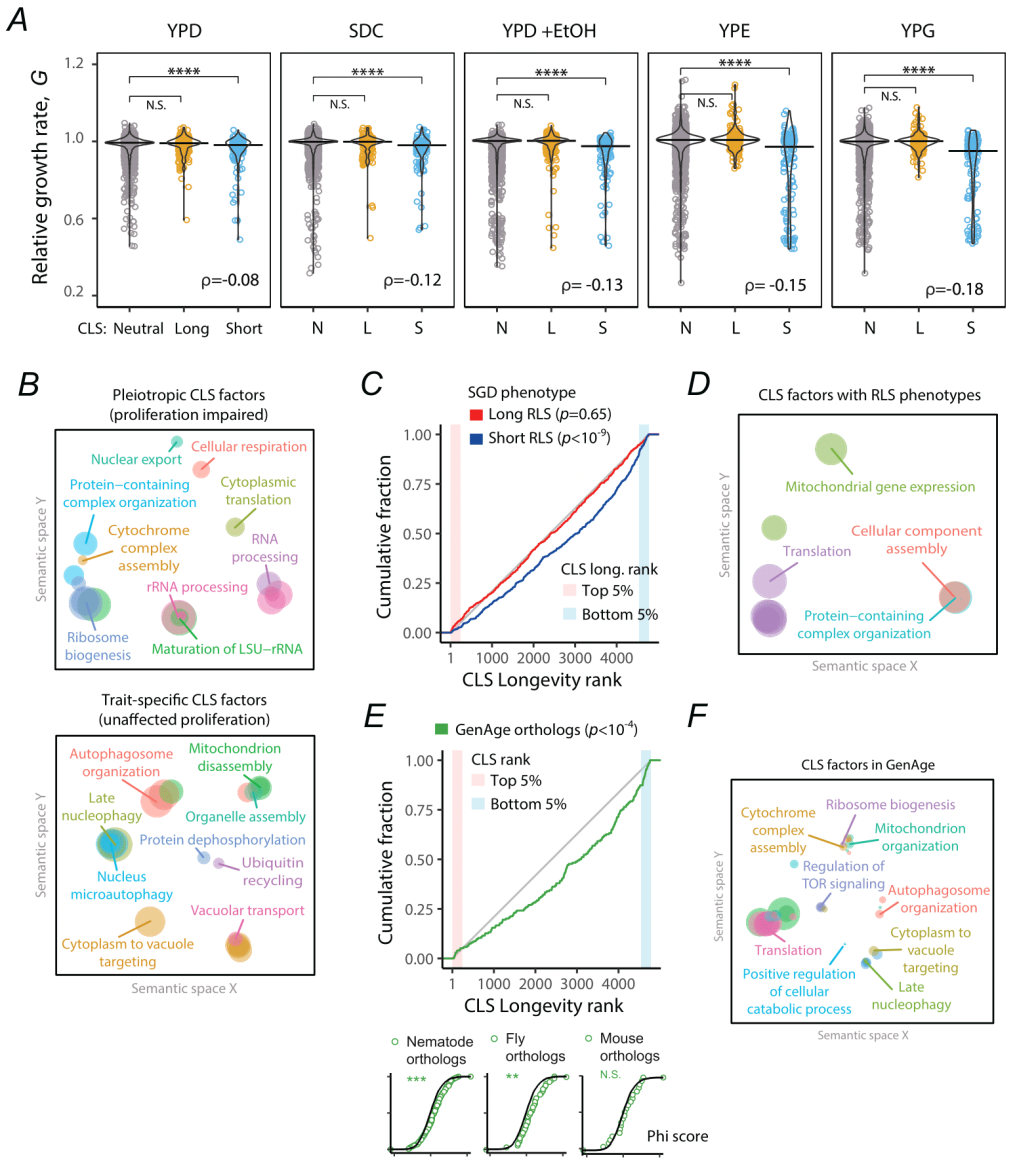
FIGURE 3: Association of core CLS factors with other phenotypes and conservation in metazoan models. **(A)** Relative growth rates of strains grouped by their inferred CLS phenotypes. Long (*n*=205) and short (*n*=123) lifespan mutants were defined as the 5% (long CLS) and 95% (short CLS) genes in the CLS longevity rank, while the rest were classified as neutral (*n*=3018) ***p<10^-3^ Wilcoxon rank sum test. For each comparison, the Spearman correlation (ρ) is between all CLS ranks and their associated growth rates. Mutants’ growth-rate data are from 23. Media, YPD is yeast peptone dextrose rich, SDC is synthetic complete dextrose, YPD+EtOH is 6% added ethanol, YPE is ethanol carbon source, and YPG is glycerol carbon source. **(B)** REVIGO visualization of GO enrichment analysis for genes in the top and bottom 5% of the CLS longevity rank. The top panel shows functional enrichment of CLS genes with growth rate defects (*G*<0.95), while the bottom panel shows genes with no detectable growth defects. **(C)** Cumulative distribution of mutants with long RLS (red, *n*=632) or short RLS (blue, *n*=452) in the CLS longevity rank, compared to a null expectation of uniform distribution shown in gray (Wilcoxon rank sum test). Shaded areas indicate the top and bottom 5% of the CLS longevity rank. **(D)** REVIGO visualization of GO enrichment analysis for genes in the top and bottom 5% of the CLS longevity rank associated with RLS phenotypes. **(E)** Cumulative distribution in the CLS rank of *C. elegans*, *D. melanogaster*, and *M. musculus* orthologs from GenAge (*n*=134, 40, and 21, respectively), compared to the null expectation of uniform distribution in the rank shown in gray (Wilcoxon rank sum test). Plots at the bottom show the cumulative distribution of orthologs *phi-score* compared to entire dataset (**p<10^-2^, ***p<10^-3^; Wilcoxon rank sum test). **(F)** REVIGO visualization of GO enrichment analysis for genes in the top and bottom 5% of the CLS longevity rank showing orthologs in the GenAge database.

Longevity phenotypes may be associated with the impairment of functions that are important for reproductive traits, in an antagonistic pleiotropic manner 36. Likewise, pleiotropic functions required for reproductive fitness and long-term survival would involve gene mutations with detrimental effects on both traits. To describe the trends in the relationship between CLS and cellular proliferation, we used quantitative growth-rate data obtained from yeast mutants growing under different conditions 37. Compared to the distribution of growth effects of mutants with no CLS phenotype, the distributions of strains with short CLS phenotypes were consistently skewed to slow-growth phenotypes (**Figure 3A**). The positive correlation between slow growth and decreased survival was higher when looking at proliferation under stress (YPD +EtOH) or respiratory conditions (YPE and YPG), compared to growth under fermentative conditions (YPD and SC). This indicates that stationary phase survival requires a set of functions that overlap with stress response and respiratory growth. These results suggest that the CLS phenotype in yeast is partly shaped by direct pleiotropy, but not typically by antagonistic pleiotropy. Yet, we note that many mutants with consistent CLS phenotypes showed no detectable growth defects, suggesting that a considerable number of genes impact postmitotic-survival phenotypes without playing major roles in cellular proliferation.

To describe which functions drive both chronological survival and proliferation in a pleiotropic or non-pleiotropic manner, we carried out a functional enrichment analysis of the cellular processes represented among gene deletions at the top and bottom of the CLS longevity rank, while also examining the overlap with effects on growth rate. Pleiotropic deletions affecting both CLS and cell proliferation phenotypes were primarily enriched in cytosolic translation and ribosome biogenesis, as well as in cellular respiration (**Figure 3B**, top panel). In contrast, non-pleiotropic CLS deletions with no impact on growth phenotypes were primarily enriched in various autophagy and protein degradation pathways (**Figure 3B**, bottom panel). Together, these results suggest that longevity regulation in yeast can occur through both general growth-related mechanisms and more specialized, post-mitotic survival pathways.

### Some CLS factors also influence the replicative lifespan of yeast

A prevailing question in yeast aging genetics is whether genes that influence CLS are also involved in RLS, and if so, which are the functions affecting both traits 38. To interrogate the associations of CLS genes with replicative aging, we retrieved a set of deletion strains with short-RLS and long-RLS phenotypes reported in SGD phenotype ontology 39. The distribution of mutants with short RLS was strongly biased to the corresponding side of the CLS longevity, although no difference was observed for long-RLS mutants (**Figure 3C**). This suggests that a common set of genes and functions are required to prevent both replicative and chronological aging in yeast. In agreement with this trend, mutants with long-RLS were marginally enriched at the top of the CLS longevity rank (p=0.0517), reflecting a common set of genes promoting both replicative and chronological longevity. The main cellular functions enriched in the set of shared replicative and chronological longevity factors were related to mitochondria, translation, and protein complexes with roles on cellular respiration, nutrient signaling, and protein degradation (**Figure 3D**; Table S3). Together, these results show that there is a specific common set of genes and functions contributing to mitotic and postmitotic survival in yeast.

### CLS factors are enriched in orthologs of aging genes in metazoans

Genetic analysis of aging in yeast ultimately seeks to serve as a paradigm to understand aging in other organisms, including humans. Replicative-longevity pathways in yeast have been shown to be conserved across eukaryotes, but whether conserved CLS pathways impact the lifespan of other model organisms has remained elusive 28,40,41. With a catalog of consistent CLS factors in hand, we asked whether CLS factors in yeast were enriched in animal orthologs associated with aging and longevity, as compiled in the GeneAge database 42. The distribution of mutants with orthologs in GenAge was biased to short-CLS longevity ranks (*p*<10^-5^, Wilcoxon rank-sum test). While no enrichment was observed in the high top 5% of the CLS longevity ranks (*p*=0.503, hypergeometric test), genes at the bottom of the longevity rank—short CLS mutants—had a higher fraction of orthologs associated with aging in three metazoan models (**Figure 2E**). We confirmed that the CLS phenotype probability distributions of the metazoan orthologs were different to the overall CLS longevity rank when looking independently at the orthologs from nematodes or flies, but not for mice (**Figure 2E**, bottom). Yeast genes in the top and bottom 5% of the CLS longevity rank with metazoan orthologs in GenAge were enriched in regulation of TOR signaling, autophagy, translation, and mitochondrial functions (**Figure 2F**). These results suggest that an enriched fraction of conserved genes underlying CLS have also conserved their aging-related functions from ascomycetes to metazoans, underscoring the potential of the yeast chronological longevity paradigm in aging research.

### An integrated view of the downstream cellular processes of CLS

**Figure 4:**
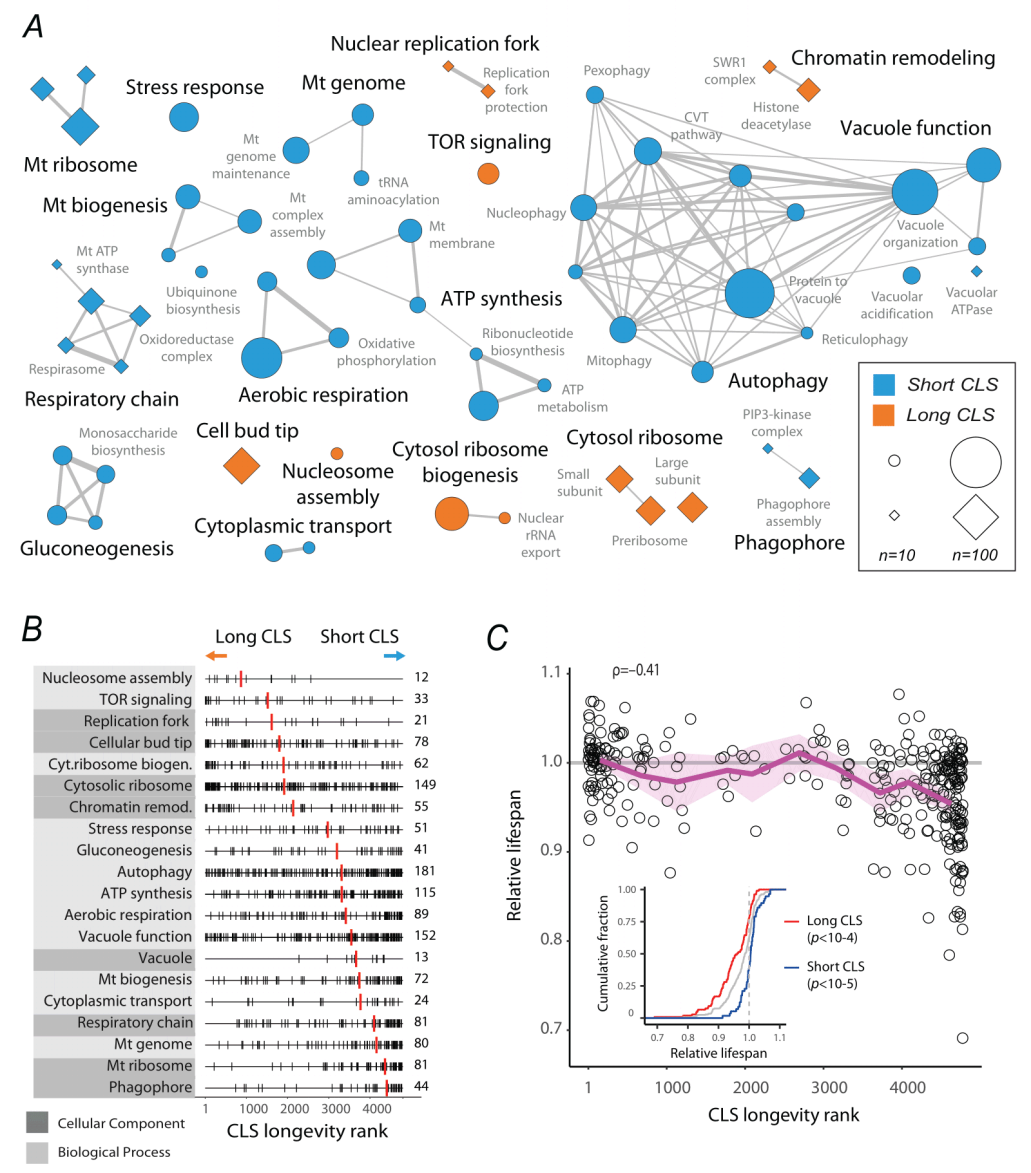
FIGURE 4: Core genetic determinants of CLS in yeast. **(A)** Network representation of functional ontologies enriched at the extremes of the CLS longevity rank, according to GSEA. Each node is a Gene Onthology Biological Process (circles) or Cellular Component (diamonds) term showing enrichment of long (orange) or short (blue) CLS ranks; node size indicates the number of genes within the GO term. Edges are the Jaccard indexes (*Ji*) indicating overlap in gene content between terms; only edges with *Ji*>0.1 are shown. The CLS clusters are labeled in bold according to the most common GO terms within the cluster (Table S4). **(B)** The rank distribution of genes in each CLS cluster along with the median gene rank of the cluster (red lines). The number of genes in each CLS cluster is shown on the right. **(C)** Plot shows the experimental characterization of 322 gene deletions representing core CLS functions identified by GSEA, plotted against the CLS longevity rank. The magenta trend line shows the decile average with confidence intervals and Spearman rank correlation (*p*<10^-14^). The inset displays the cumulative distribution of relative lifespans measured for genes in the top (red) and bottom 5% (blue) of the CLS longevity rank, compared to all 322 genes (gray line; Wilcoxon rank sum test).

Our ranked catalog of aging factors allowed us to inquire which functions and pathways influence the CLS of yeast cells in a robust manner, independently of the experimental conditions used. To this end, we performed Gene Set Enrichment Analysis (GSEA) 43 using the *phi-score* distribution of the CLS longevity rank. Enriched biological processes and cellular component ontologies were further clustered based on their shared gene content, portraying the main functions associated with CLS in yeast (**Figure 4A**; Table S4).

Functional analysis showed that, in yeast, respiratory function, biogenesis of mitochondria, the ATP-synthesis machinery, autophagy and different kinds of selective autophagy, other processes involving vacuolar function, transport in the endoplasmic reticulum, response to stress, and gluconeogenesis were all consistently required for postmitotic survival, preventing premature chronological aging. At the opposite side of the CLS phenotype spectrum, genetic perturbations in TOR signaling, replication of DNA, protein complexes at the cell-bud tip, biogenesis of cytosolic ribosomes, and some chromatin-organization complexes usually resulted in increased chronological longevity. We observed that the resulting functional clusters associated with CLS were usually composed of genes with a wide range of phenotypes (**Figure 4B**). For instance, the cytosolic ribosome gene set was enriched at the long-CLS extreme of the spectrum but also included a considerable number of genes at the opposite end of the short-lived phenotype distribution. Likewise, autophagy and vacuole function were gene sets enriched in short-lived phenotypes, but also showing genes in the top longevity ranks.

As an additional validation of the ensuing integrated CLS longevity rank, we experimentally measured the relative lifespan of 322 gene-deletion strains of genes representative of the resulting enriched functions, associated with different ranks (Table S5). To this end, we conducted competitive CLS assays of this set of prototrophic gene deletions aged in deep-well plates with unbuffered SC 2% glucose medium (see Methods). As expected, the relative lifespans were negatively correlated with the CLS longevity rank (**Figure 4C**). Moreover, those gene deletions in the top 5% of the longevity rank were significantly long-lived when assayed experimentally, while those in the bottom 5% were usually short-lived. These results further suggest that our integrated CLS rank informs on consistent genetic aging factors, regardless of the experimental condition used.

We note that our meta-analysis-based approach to identifying consistent factors underlying CLS provides a more comprehensive functional perspective than analyses of individual datasets alone. Specifically, we observed limited overlap among the GSEA hits from individual screens (Figure S5). For example, short-lived deletions affecting mitochondrial genome translation and significantly enriched in aerobic respiration were only detected in three out of the nine datasets, yet clearly emerged in the integrated analysis (**Figure 4A**). A similar pattern was observed for long-lived deletions related to autophagy, which appeared as hits in only four datasets. In fact, two datasets showed no functional enrichment whatsoever when individually analyzed (Figure S5). Notably, this GSEA analysis also highlights functions specific to certain experimental setups, that is, those appearing in individual datasets but not in the integrated functional network. For instance, pheromone-response signaling pathways were detected only in the ‘Matecic’ and ‘Gresham-L’ datasets, while endocytosis appeared solely in the ‘Gresham-P’ dataset. Together, our functional analysis of consistent CLS factors provides an integrated view of the downstream mechanisms mediating chronological aging in yeast cells.

### Potential transcriptional regulators of CLS

**Figure 5:**
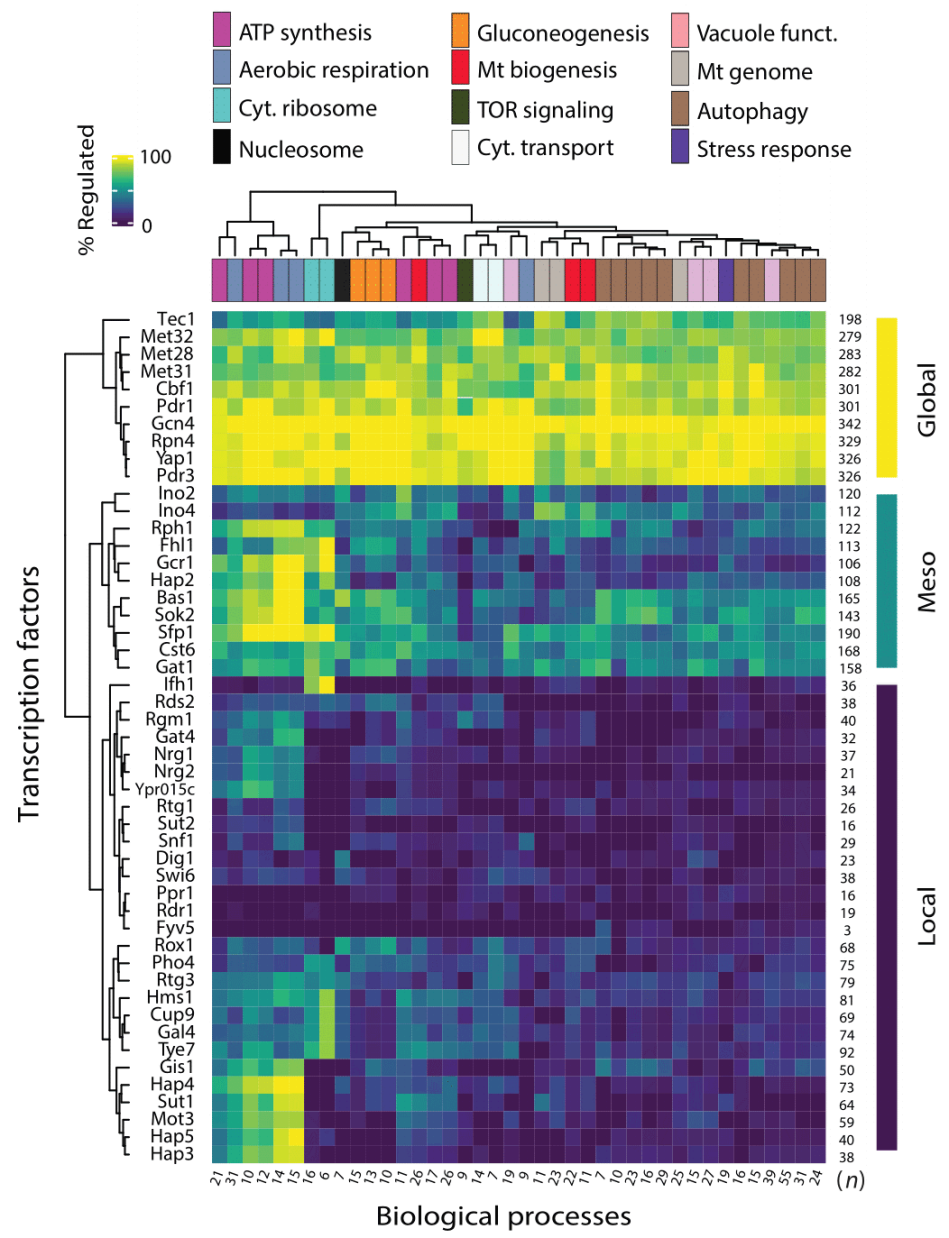
FIGURE 5: Potential transcriptional regulators of core CLS genetic factors. Heatmap showing the fraction of leading-edge genes within each biological process (columns) predicted to be regulated by each transcription factor (rows), based on TFRank analysis. Functional groups are those resulting from the GSEA analysis shown in Figure 4. Rows and columns are ordered by hierarchical clustering of regulatory profiles. Transcription factors were defined as ‘global’, ‘meso’, or ‘local’ regulators based on the patterns of leading-edge genes regulated. The number of leading-edge genes in each functional group and associated to each transcription factor are shown below and at the right, respectively.

To provide a regulatory picture of chronological longevity, we explored which transcription factors (TFs) operate upstream of the identified CLS factors. Specifically, we used the TFRank algorithm 44 to obtain the transcriptional regulators of the leading-edge genes—those that contribute most to the observed GSEA results—within all GO terms of Biological Process CLS clusters (345 genes in total, see Methods). Forty-nine TFs were the main regulators of the defined set of genes consistently associated with CLS phenotypes (**Figure 5**; Table S6). At the phenotypic level, we observed that the individual deletions in this set of potential CLS regulators showed a wide range of CLS effects, with no significant changes in their median effect compared to the overall CLS longevity rank (*p*=0.73, KS test). These TFs included some known positive and negative regulators of CLS, such as Bas1 12, Tec1 31, Gis1 12, and several subunits of the HAP heteromeric complex 13. However, we note that most of this putative set of CLS regulators had not yet been described in terms of their possible contribution to chronological aging and longevity.

To obtain a broader picture of the regulatory landscape of chronological aging in yeast, we clustered the TF hits and the CLS biological processes based on the fraction of leading-edge genes regulated by each TF in each GO term (**Figure 5**). This analysis revealed a subset of highly pleiotropic TFs connected to most genes across multiple functional clusters, which we termed ‘global’ CLS regulators. For example, Gcn4, Met28, Met31, Met32, and Cbf1 emerged as global regulators, likely through their control of genes involved in amino acid biosynthesis and sulfur metabolism. Tec1, a regulator implicated in the MAPK filamentous-growth response and Ty1 retrotransposon expression, also showed widespread regulatory associations across nearly all functional clusters—though often at lower frequencies—further supporting its role as a global regulator. In contrast, several TFs showed more restricted associations with specific clusters; for instance, Hap3, Hap4, and Hap5—key transcriptional activators of aerobic respiration and ATP synthesis—functioned as ‘local’ regulators of those specific CLS functional clusters. At an intermediate level of regulatory breadth, we identified a group of TFs linked to genes involved in diverse processes. These ‘meso’ regulators showed the strongest connections to mitochondrial biogenesis and autophagy clusters, with more limited but consistent involvement in other functions. Overall, these findings offer a comprehensive view of the transcriptional regulatory architecture potentially orchestrating chronological aging and longevity in budding yeast.

## DISCUSSION

The inherent simplicity of the chronological longevity paradigm, combined with the vast genetic toolbox available in yeast, would make this a straightforward model for studying the genetic underpinnings of cellular aging and longevity in a more systematic manner. However, a comprehensive understanding of the CLS phenotype has proven to be challenging. For instance, Smith *et al*. observed that only 22 CLS hits were shared among three genome-wide aging deletion screens 32. With the larger set of datasets now available 24,25,26,27,28,29,30,31, we used a meta-analysis approach to reveal mutants that are robustly associated with chronological aging in yeast.

PROMETHEE enabled the integration of heterogeneous datasets from different experimental approaches into a single outranking analysis, providing a transparent way to prioritize genes and processes and to identify CLS factors robust across diverse conditions. Nevertheless, we acknowledge that the method has limitations 40. PROMETHEE does not account for the statistical uncertainty inherent in large-scale screen datasets, and the extent to which rank reversal might occur when applied to these datasets was not systematically addressed. In fact, the rankings were somewhat sensitive to the chosen criteria weights, and the computational demands of extensive pairwise comparisons limited the consideration of all possible datasets, which could affect the stability and coverage of the rankings. Despite these constraints, the resulting integrated information aligned well with smaller-scale CLS phenotypes and our own experimental CLS phenotyping of over 300 gene deletion strains, suggesting that it provides valuable insights into the genetic and functional basis of chronological aging in yeast.

Importantly, most of the ensuing consistent downstream cellular mechanisms of chronological longevity in yeast turned out to be widely recognized hallmarks of aging 46. Specifically, mitochondrial function, autophagy, the replication fork involved in genome stability, the protein-synthesis machinery, along with nutrient-sensing and epigenetic regulators, conformed most of the integrated functional landscape underlying CLS. At the same time, our integrated analysis also highlights functions and processes that have been less widely recognized in aging research, such as histone exchange mediated by the SWR1 complex, involvement of the replication fork, and selective autophagy targeting the nucleus (nucleophagy) and peroxisomes (pexophagy). The chronological aging paradigm thus offers an untapped resource to systematically inquire how these universal hallmarks of aging or more yeast-specific factors operating at the cellular level are integrated with one another. For instance, Synthetic Genetic Array technology 47, combined with high-resolution CLS profiling to detect lifespan-epistasis interactions 48, could provide a relatively straightforward approach to comprehensively dissect the complex genetic landscape of aging cells.

It must be noted that, even though our meta-analysis pointed to cellular processes robustly associated with aging, it still holds that the phenotypic context of chronological aging is highly variable. Our results not only confirmed a general lack of overlap in CLS phenotypes across assays 32 but also showed that even within the most relevant functional clusters there was wide variation within the CLS longevity ranking. For instance, we observed that varying only one component of the aging media but maintaining the same genetic background and experimental setup—such as in ‘Gresham-L’ vs ‘Gresham-P’ and ‘Campos-NR’ vs ‘Campos-DR’ datasets—led to highly significant yet not fully correlated outcomes (ρ=0.11 and ρ =0.54, respectively). These extrinsic factors, along with cell-intrinsic factors such as ploidy or genetic background, may have a strong impact on the phenotypic outcomes 49,50,51,52,53. Moreover, random variation in lifespan is a persistent feature even in laboratory assays where genotype and environment are tightly controlled, as demonstrated in yeast, fly, and nematode models 54,55,56. It is likely that the extent to which each of these contextual factors impacts CLS outcomes can only be effectively addressed on a smaller scale. Yet, as with many other areas of genomics, large-scale analyses are likely to reveal useful patterns and provide integrated knowledge, contributing to a deeper mechanistic understanding of chronological longevity, as demonstrated here.

Long-lived rather than short-lived phenotypes are arguably more informative about the underlying mechanisms of aging 10,57. Our meta-analysis revealed cellular processes and signaling pathways whose inactivation or alteration consistently resulted in increased CLS. For instance, deletion of genes coding for ribosomal proteins or ribosome biogenesis and other processes related to cytosolic translation were linked to increased CLS. This was consistent with strong experimental evidence that ribosome regulation modifies not only yeast CLS and RLS 58,59, but also the lifespan of other model organisms. RNAi treatment directed to ribosomal genes and translational regulators increases the lifespan of *Caenorhabditis elegans* 60,61, while mutation of upstream regulators of translation such as the ribosomal-protein S6 kinase and 4E-BP impacts lifespan in *Drosophila melanogaster* 62,63. Deletion of the ribosomal protein s6 has also been shown to increase lifespan in mice 64. Our results also showed that, in yeast, a major limiting factor of chronological longevity involves a widely conserved chromatin remodeling complex involved in nucleosome sliding and exchange of variant histones 65. Previously, the Swc3 subunit of the SWR1C complex was recognized as a potential pro-aging factor in a microarray-based genome-wide CLS screen 25. Loss of several subunits of this complex mimics the effects of longevity by dietary restriction 30. Our meta-analysis further indicates that SWR1 is a robust aging modulator, whereby its genetic inactivation results in consistently increased CLS. Yet the cellular mechanisms by which the conserved histone-exchange Swr1 complex restricts chronological longevity remain unknown.

The distribution of genes in the CLS longevity rank suggests that the two ways in which yeast cells age—namely mitotically and post-mitotically—share common mechanisms. Specifically, we observed an enrichment of short-RLS associated genes at the short-lived extreme of the CLS spectrum, while long-RLS deletions were marginally enriched at the long-lived side of CLS distribution. To the best of our knowledge, this level of genetic association between RLS and CLS had not been previously observed globally. Substantial efforts are being directed to gain a comprehensive dataset of RLS phenotypes (e.g. 66,67), which will ultimately allow a more thorough assessment of the functional relationships of this model of mitotic aging with CLS. We also found that robust aging phenotypes were more common in yeast genes with orthologs related to aging and longevity in metazoan models, albeit with a weaker signal and only for short-lived effects. We note that this could be in part due to the observed direct pleiotropy nature of many CLS genes, since genes with a high contribution to fitness tend to be conserved across organisms 68.

Our study also provides an initial view of the transcriptional regulatory architecture that may underlie chronological aging and longevity in yeast. Notably, many of the TFs identified in this analysis have not been previously linked to CLS regulation, highlighting promising candidates for future investigation. For instance, our findings underscore a broader role for MAPK signaling pathways in the regulation of aging in yeast, suggesting previously unrecognized connections. In this regard, the Tec1 regulator operating downstream of MAPK signaling is a known positive regulator of yeast CLS 31 and the stability of this transcriptional activator is regulated by TORC1, indicating that Tec1 links TORC1 and MAPK signaling in response to different stimuli 69,70. Previously, Aluru *et al*. revealed an unexpected role in aging of Fus3 and Kss1 involved in the pheromone and filamentation pathways, whereby deleting these MAPKs leads to increased CLS 71. Authors speculated that downstream autophagy activity underlies this phenotype along with the observed interactions with TOR1-signaling. In a previous study, our group showed that the activity of the Ste12 transcriptional regulator of the pheromone and filamentation pathways promotes chronological longevity and that *STE12* overexpression is enough to extend CLS 31. In these studies, authors have speculated roles for autophagy and cell-cycle arrest possibly operating downstream of Ste12 and Tec1. It is also worth noting that Tec1 is the main transcriptional regulator of retrotransposable Ty1 elements 72,73. Ty1 activity promotes chronological longevity 74, which raises the possibility that the dynamics of these mobile genetic elements may also partly account for the effects associated with Tec1 regulation.

This study highlights the main mechanisms of chronological aging in yeast, which shares many of the widely recognized universal underpinnings of aging. Moreover, the genetic relationships between RLS and CLS, along with the evolutionary and functional conservation of some aging genes from yeast to animals, underscore the potential use of the amenable yeast CLS model in aging research. By fully exploiting the vast genetic toolbox in yeast, the chronological longevity paradigm will provide further insights into the genetic logic of aging and the genetic wiring of aging cells.

## MATERIALS AND METHODS

### Strains and media

Starter *de novo* *ho*Δ deletion strains were generated by PCR-based direct gene replacement using natMX4 cassette from the pAG25 plasmid on the YEG01-RFP and YEG01-CFP parental strains (*MAT*α *PDC1-XFP-CaURA3MX4* *can1*Δ:*STE2pr-SpHIS5* *lyp1*Δ *his3*Δ1 *ura3*Δ0 *LEU2*) 30. Mutants used for competitive aging screening, were derived from crossing the starter *ho*Δ strains to selected mutants of the yeast deletion collection (*MAT*a BY4741) using the Synthetic Genetic Array methodology 47,75. Diploids were sporulated and subjected to three rounds of haploid selection (*HIS^+^* for *MATa* mating type, *URA^+^* for the fluorescence marker, and *G418^+^* and NAT*^+^* for knockout selection). The *ho*Δ* his3*Δ double neutral insertion was defined as the WT strain for competitive-aging assay.

Synthetic Complete (SC) medium used for CLS aging assay contained 6.7 g/L yeast nitrogen base (YNB) without amino acids (Difco 291940), 20 g/L glucose, and 2 g/L amino acid supplement mix (Yeast Synthetic Drop-out Medium Supplements without uracil, Sigma Y1501, plus 0.076 g/L Uracil). Low fluorescence medium (YNB-lf) for outgrowth cultures was prepared as described 76.

### Competitive-aging CLS assay

High throughput fluorescence-based CLS profiling was based on Avelar-Rivas *et al. *(2020) 48. In brief, saturated cultures of the selected strains were pinned to 96-well plates with 150 μL of fresh SC medium. These plates were left to saturate for 48 h at 30°C without shaking. Saturated RFP-labeled mutants and CFP-labeled wildtype (WT) cultures were mixed in 2:1 RFP:CFP ratio; reference wells with WT_RFP_ or WT_CFP_ strains only were included, for background fluorescence measurements. Mixed cultures were pinned to 96-well deep wells containing 700 μL of SC medium and grown to saturation at 30°C and 70% relative humidity, without shaking. After four days and at daily intervals afterwards, cultures were resuspended by shaking and 5 μL aliquots were inoculated with an automated robotic pipetting arm into 150 μL of fresh YNB-lf medium. In these outgrowth cultures, OD_600_ and fluorescence (RFP and CFP) were measured every 2 h until saturation, with a Tecan Infinite M1000 plate reader. Sampling was repeated for six days in total. Optimal gain values were 164 and 204 for CFP and RFP signal, respectively; these were obtained from a previous outgrowth at late exponential growth, when the signal was at its maximum.

### Data analysis for competition-based CLS screening

Death rates for the fluorescence-based assay were calculated following Avelar-Rivas *et al. *2020 48. The RFP/CFP ratio was used to estimate the number of cells tagged with each fluorescence protein. Background fluorescence was subtracted for all measurements. For each sampling day (*T_i_*, in days) the outgrowth cultures measurements (*t_j,_* in hours) were compiled into a signal ratio* ln(RFP/CFP)T_i,tj_* for each sample (w) and fitted to the linear model *A_w_* + *S_w_*⋅*T_i_* + *G_w_*⋅*t_j_* + *C_Ti_*,*t_j_*, where *A* was the viable cell ratio at the start of the experiment, *G* was the growth rate difference of both strains, and *S* was the survival of the mutant relative to the WT reference. An additional term, *C_Ti_, t_j_* , was included to model the systematic variation of each plate at each stationary-phase sampling point *T_i_t_j_*. *S*=0 and *G*=0 was assumed for all WT_RFP_/WT_CFP_ competitions. The resulting set of equations was solved for *Sw* by multiple linear regression. Relative survival (*Sw*) was transformed to relative lifespan values (*Lw*), where *Lw*= 1+*Sw*. Relative lifespan for 322 out of 330 tested is provided in Table S5.

### CLS datasets

Genome-wide chronological lifespan assay data was retrieved for each publication mentioned in **Table 1** 24,25,26,27,28,29,30,31; where the experimental setup of each dataset is also indicated. In some publications, more than one medium was used for measuring the lifespan of a given knockout mutation set. In such cases, each condition was assigned as a different dataset and was analyzed separately. To integrate all high-throughput experiments into a summarized table, for every dataset a single representative lifespan value of each knockout mutant was taken. Details of data transformation for each dataset are available as Extended Methods (File S1). This process resulted in nine different datasets which can be found in Table S1. Linear modelling of correlation between datasets and experimental setup was performed using the *stats* package in R.

**Table 1:** Datasets used from gene-deletion screens of CLS phenotypes in *S. cerevisiae* ^1^. ^1 ^Datasets from the same publication using different aging media are listed as separate entries. ^a^ The number of short-CLS and long-CLS mutants are indicated here by the cut-off criteria specified in each publication.

**Dataset Name**	**Aging medium**	**Experimental setup**	**CLS assay**	**Strain, ploidy & auxotrophy**	**Query genes**	**Long/Short CLS ^a^**	**Ref.**
Powers	SC, 2% glucose, 4x Leu, Ura & His, non-buffered	200 µL monoculture, non-aerated	Microculture outgrowth	BY4743 2n (*MATa/α his3 leu2 ura3*)	4759	90 / 300	24
Matecic	SC, 2% glucose, non-buffered	10 mL pooled culture, aerated	Culture outgrowth, barcode microarray hybridization	BY4741 1n (*MATa his3 leu2 ura3 met15*)	3428	40 / 117	25
Fabrizio	SC, 2% glucose, 4x Trp, Leu, Ura & His, non-buffered	50 mL pooled culture, aerated	Culture outgrowth, barcode microarray hybridization	BY4741 1n (*MATa his3 leu2 ura3 met15*)	4112	42 / 594	26
Gresham-L	SC, 1% glucose, Leu-limited, buffered	400 mL continuous pooled culture, aerated	Culture outgrowth, barcode sequencing	BY4742 1n (*MATα his3 leu2 ura3 lys2*)	4222	458 / 3500	27
Gresham-P	SC, 1% glucose, PO_4_-limited, buffered	400 mL continuous pooled culture, aerated	Culture outgrowth, barcode sequencing	BY4742 1n (*MATα his3 leu2 ura3 lys2*)	4142	377 / 956	27
Marek	Water	200 µL monoculture, non-aerated	Microculture outgrowth	BY4741 1n (*MATa his3 leu2 ura3 met15*)	3673	58 / 566	28
Garay	SC, 2% glucose, buffered	700 µL two-strain culture, non-aerated	Microculture outgrowth, relative fluorescence	BY4741 1n (*MATa*)	3878	247 / 516	30
Campos-NR	SC Gln, 2% glucose, non-buffered	700 µL two-strain culture, non-aerated	Microculture outgrowth, relative fluorescence	BY4741 1n (*MATa*)	3717	254 / 573	31
Campos-DR	SC GABA, 2% glucose, non-buffered	700 µL two-strain culture, non-aerated	Microculture outgrowth, relative fluorescence	BY4741 1n (*MATa*)	3717	228 / 510	31

### Dataset evaluation and AUC-ROC score

A curated knockout strains list with a known confirmed phenotype in CLS was generated to be used as reference to evaluate dataset performance (Table S2). An initial list was obtained from the Yeast Phenotype Ontology at the Saccharomyces Genome Database, retrieved in June 2025 35 (https://yeastgenome.org/ ontology/phenotype/ypo). The above-mentioned list was generated by filtering the phenotype ontology for "Chronological lifespan increased" or "Chronological lifespan decreased" annotations, plus the "classical genetics" annotation. Further manual inspection of the records was done to select those mutants whose phenotype was validated by using the colony forming unit (CFU) method or live-dead staining assay. Mutants were retrieved regardless of reported culture media and conditions. When mutants were reported with opposite phenotypes—both long- and short-lived—the final phenotype designation was made on a case-by-case basis, considering the frequency of each reported outcome. In cases where evidence for opposing phenotypes was equally balanced, the mutant was excluded from the analysis (nine out of 171 cases).

To assess the CLS datasets replicability, each dataset was evaluated on their performance to segregate mutants of known phenotypes in two groups: long-lived and short-lived. With the curated mutant list, a set of true labels were generated for a binary classifier system. While testing each dataset, curated mutants were excluded when the small-scale phenotypes were validated within the same study, to avoid performance overestimation due to common source bias (Table S2). A Receiver Operating Characteristic curve (ROC) was used to evaluate the performance of each CLS dataset, using the *ROCR* package in *R* 77.

### PROMETHEE method for multivariate analysis

To generate a ranking of which gene deletions have been consistently reported as long-lived, the multiple-criteria decision analysis PROMETHEE II method was used 35, which was implemented in the *RMCriteria* package in *R* (version 4.3.0) 78. The CLS datasets ‘Matecic’, ‘Marek’, ‘Campos-DR’, ‘Garay’, and ‘Gresham-L’ were set as ranking criteria within the method. All genes with available data in at least one dataset were used as alternatives. For PROMETHEE II analysis settings, a usual preference function was applied to maximize all criteria. A final ranking was obtained using equivalent weights for each of the five datasets (w=0.2). Alternative rankings were obtained using weights proportional to area under ROC curve. (w_1_=0.2214, w_2_=0.2133, w_3_=0.1982, w_4_=0.1870, w_5_=0.1801) or priority weighting (w_1_=0.4567, w_2_=0.2567, w_3_=0.1567, w_4_=0.09, w_5_=0.04) (Table S3; Figure S4). In each case, net preference flow after PROMETHEE II analysis was used to generate the ordinal ranking and reported as *phi-score*s.

### Other phenotypic datasets

Growth rates from single knockout mutants under a variety of culture conditions were retrieved from Qian *et al*. 37. RLS data List of genes with an effect over replicative lifespan was retrieved from the Yeast Phenotype Ontology at the Saccharomyces Genome Database 39. The records labeled under "increased replicative lifespan" and "decreased replicative lifespan" were retrieved and filtered to select knockout mutations and microdissection RLS methods. Genes associated to lifespan modulation in mouse (*Mus musculus*), fruit fly (*Drosophila melanogaster*), and nematode (*Caenorhabditis elegans*) were retrieved from the GenAge database (https://genomics.senescence.info/genes/models. html) 42. Genes were included regardless of their short or long lifespan effects or the genetic perturbation used for phenotyping. Yeast orthologs of these genes were also retrieved from GenAge.

### GO enrichment analysis

Gene Ontology enrichment analysis for phenotypic datasets was made using the *clusterProfiler* package in *R *79. Biological Process Gene Ontology annotation was sourced from the org.Sc.sgd.db package (v.3.18) (doi: 10.18129/B9.bioc.org.Sc.sgd.db). p-values were adjusted using Benjamini-Hochberg method and adjusted *p*<0.05 were regarded as enriched. REVIGO implemented in the R package *rrvgo* 80 was used to summarize and visualize the enriched GO terms by removing redundancy and grouping semantically similar terms into representative categories.

### Gene Set Enrichment Analysis

Functional enrichment analysis of the final ranked list of robust lifespan factors and CLS datasets was done using the Gene Set Enrichment Analysis (GSEA) method implemented on *clusterProfiler* package in *R* 43,79. GO term annotations were retrieved from org.Sc.sgd.db package (v.3.18). Only GO sets with 10-100 genes were considered for GSEA. In each case, GO terms with FDR-adjusted *p*< 0.05 were regarded as enriched. For the CLS datasets, the representative lifespan values for each mutant (Table S1) were used as input, and enrichment calculated only for Biological Process (BP) terms. For the final ranking set, *hi-scores* derived from PROMETHEE II analysis were used as input for gene list ranking. Both BP and Cellular Component (CC) GO terms were assessed as gene sets for enrichment. The Jaccard index was calculated for comparing the gene overlap among the enriched sets. Hierarchical clustering of the resulting Jaccard indexes was done to identify redundant or similar GO terms. Assignation of GO terms to biological clusters was based on this clustering, using arbitrary names. Full and revisited list of enriched gene sets for final ranked list, along with functional clusters are provided in Table S4.

### TFRank analysis

Prediction of the main transcriptional factors regulating CLS consistent biological processes was made using the TFRank approach (http://www.yeastract.com/formrankbytf.php 44. Leading Edge genes of enriched GO terms of GSEA in the BP category were used as input (Table S4). Settings were set to check for all TFs available in YEASTRACT and filter documented regulations by DNA binding or expression evidence. No filtering by environmental conditions was added. TFs were considered of relevance if *p*<0.01. Significant TFs, their targets within leading edge genes, and percentage of genes regulated are provided in Table S5.

## CONFLICT OF INTEREST

The authors declare that they have no conflict of interest.

## SUPPLEMENTAL MATERIAL

Click here for supplemental data file.

Click here for supplemental data file.

Click here for supplemental data file.

Click here for supplemental data file.

Click here for supplemental data file.

Click here for supplemental data file.

Click here for supplemental data file.

All supplemental data for this article are available online at www.microbialcell.com/researcharticles/2025a-cruz-bonilla-microbial-cell/.
