# Supplementary material for: The core genetic drivers of chronological aging in yeast are universal regulators of longevity
Source: Microb Cell. 2025 Oct 31;12:274–89. doi: 10.15698/mic2025.10.861 (PMC12632094; doi:10.15698/mic2025.10.861)
Supplement: Supplementary file 1 [file mic-12-274-s01.pdf]

Figure S1

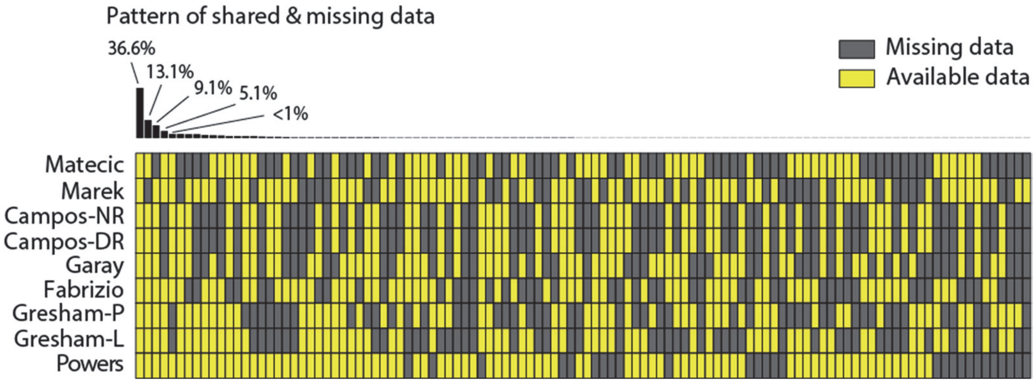

Supplementary Figure S1. Comparison of data available on the high-throughput CLS datasets. Data availability patterns of knockout mutants are shown for all datasets, except 'Burtner' due to the large fraction of missing data. The most comprehensive dataset was 'Powers' (1.7% missing genes), while the least complete were 'Gresham-P' with 13.1% and 'Matecic' with 9.1% missing genes. Gene availability patterns are labeled with their corresponding percentage; patterns with no percentage shown account together for ~13% of occurrence patterns, but each with less than 1% occurrence.

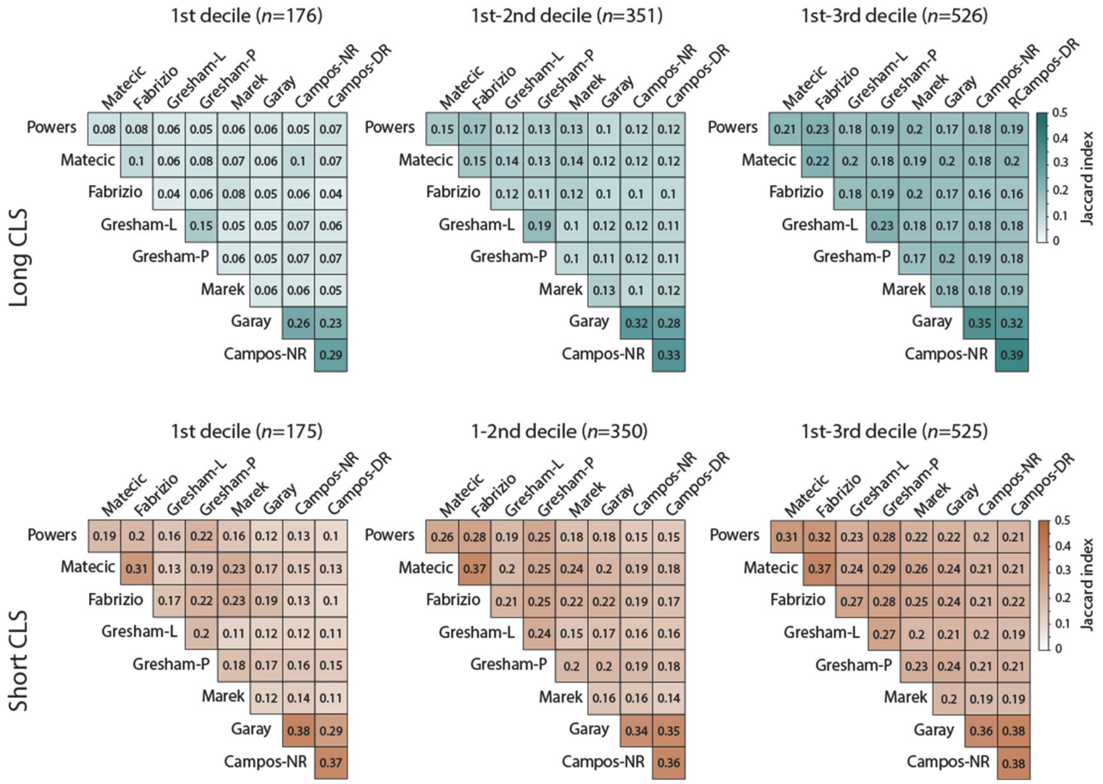

Supplementary Figure S2. Overlap similarity among CLS datasets. Each ranked list of mutants, from highest to lowest lifespan according to each assay measurements, was divided into deciles. Only the shared gene set among all datasets is considered in this comparison (n=1751). The first three deciles were assigned as long-lived mutants, while the last three were defined as short-lived. Overlap among dataset deciles was estimated with the Jaccard similarity index. The number of genes in each decile comparison is shown.

Figure S3

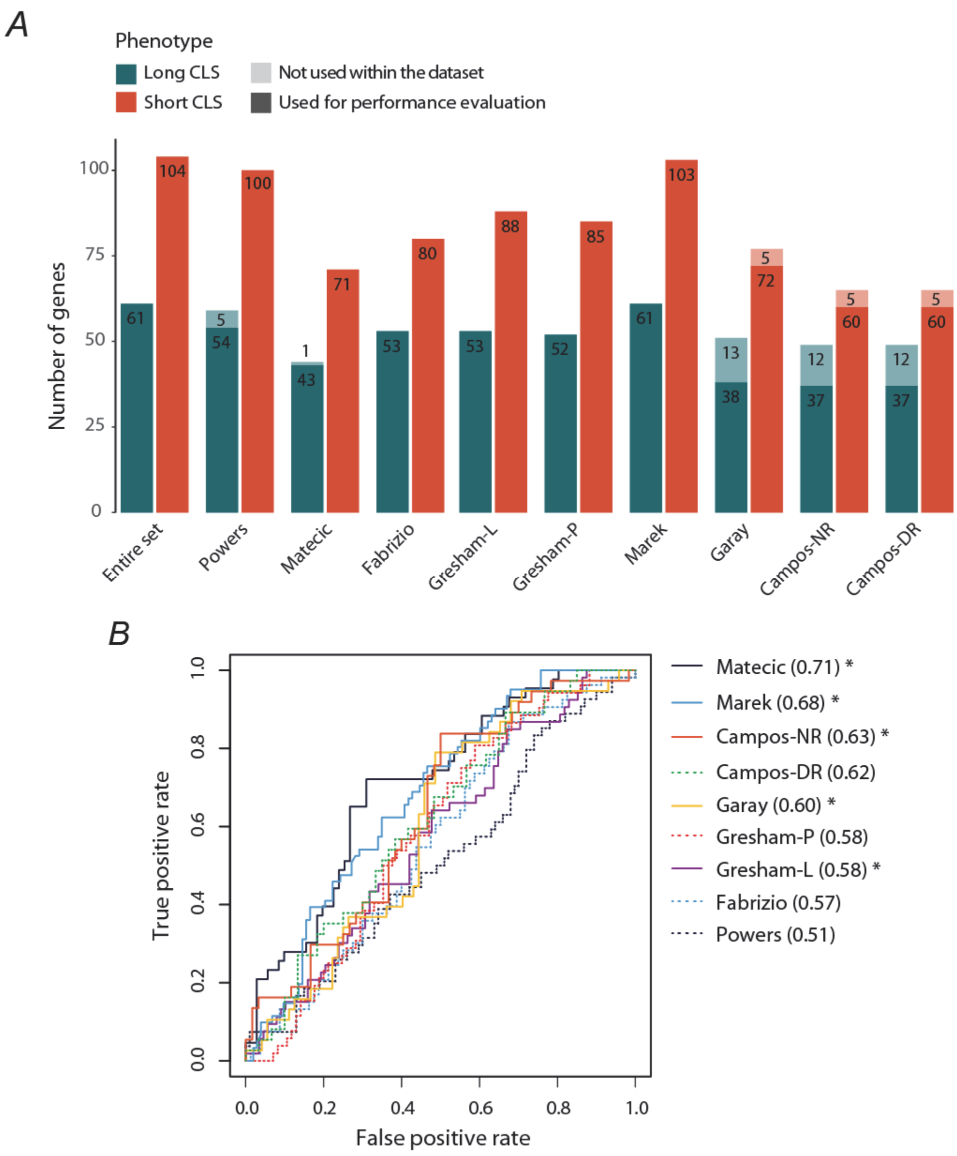

Supplementary Figure S3. Evaluation of CLS datasets on AUC-ROC curves. **A)** Availability of the curated CLS phenotype set used to evaluate the datasets. Mutants are separated into long- and short-lived phenotype. The curated set of 171 deletions with CLS phenotypes is provided in Table S2. We note that the curated CLS phenotype set includes mutants that were validated at the low scale in some of the large-scale screens evaluated (light background in bars). The final number of mutants used for ROC evaluation after common source removal is shown in solid background. **B)** ROC curves of high-throughput CLS datasets against the curated CLS phenotype set. The area under curve (AUC) is shown beside each dataset. Datasets selected for the final ranked list of robust CLS factors are indicated with asterisks and shown with solid lines.

Figure S4

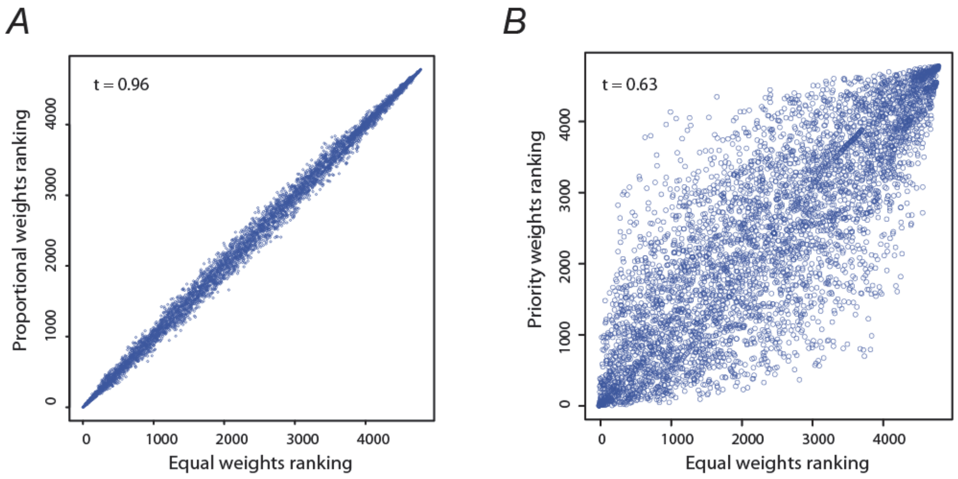

Supplementary Figure S4. Comparison of alternative rankings obtained using different weight allocations for the CLS datasets on the PROMETHEE II method. Ranking with original weight allocation is labeled here Equal. We generated two alternative rankings with new weight distributions, labelled Proportional and Priority. Kendall tau correlation is shown in each comparison between **A)** equal vs proportional and **B)** equal vs priority weights rankings.

Figure S5

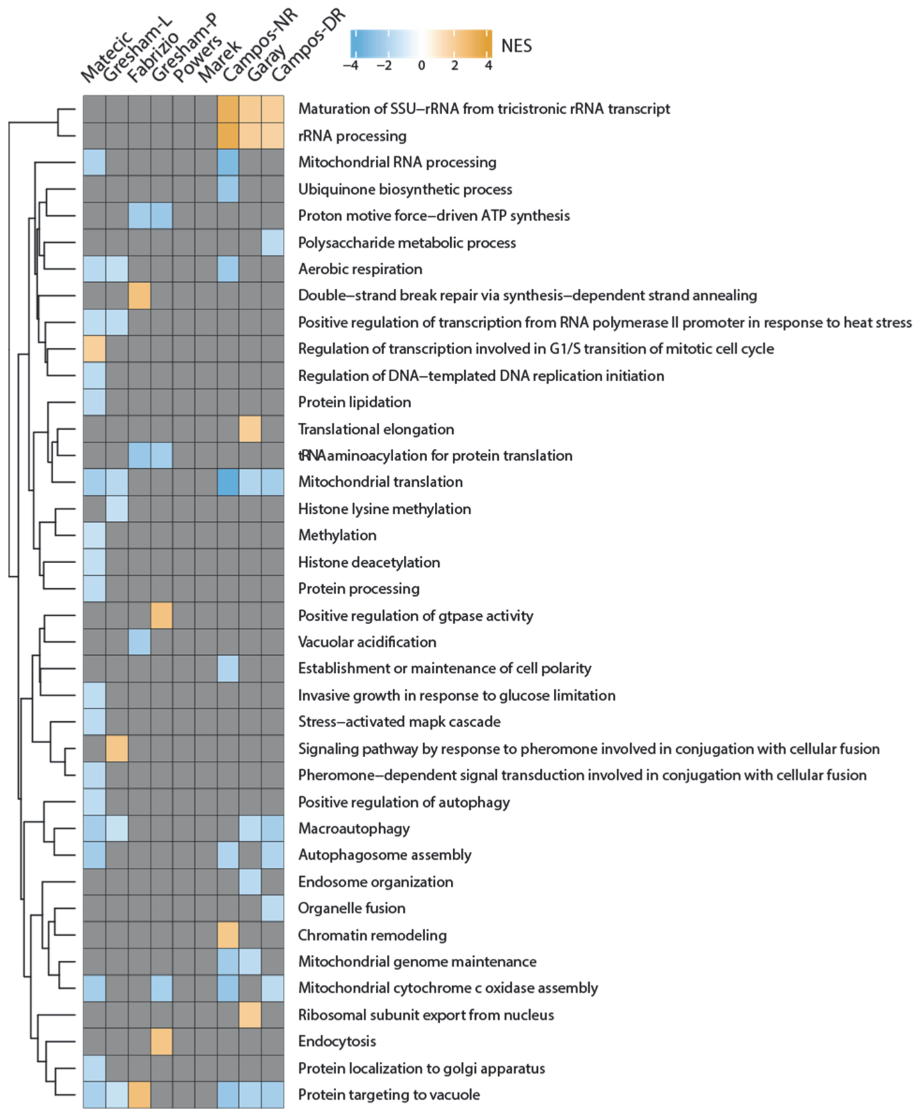

Supplementary Figure S5. GSEA analysis results of individual CLS datasets. Enriched biological processes in each dataset were assessed applying GSEA over the representative lifespan values of each transformed dataset (Table S1). Significant GO term list ($p < 0.05$) was further reduced for visualization by semantic similitude analysis using REVIGO (Jiang distance, similarity threshold 0.7). Normalized Enriched Scores (NES) indicate whether GO term is enriched within the dataset in mutants with long CLS (NES>0) or short CLS (NES<0). Grey areas indicate no GO term enrichment in the particular dataset. Dataset 'Marek' showed enrichment in few GO terms before reducing for visualization, 'Powers' showed no enriched terms. Hierarchical clustering of GO terms and datasets was made according to semantic distance and functional profile similarity, respectively.

Extended Methods

Chronological lifespan datasets and data transformation. To integrate all high-throughput experiments into a summarized table, for every dataset a single representative lifespan value of each knockout mutant was taken. When data was a single value describing lifespan, the data was taken as reported, otherwise, data was transformed as follows:

- ‘Powers’: Original data was the relative viability of knockout mutant at weeks 1,2,5,7. The representative CLS value was the sum of the relative viability across measurements. Reference [1].
- ‘Matecic’: Original data was the log2 signal ratio of both up and down hybridization tags measured at days 9, 21 and 33, and relative to day 1. The representative CLS value was the mean area under curve of the up and down hybridization tags signal ratio curves. Reference [2].
- ‘Fabrizio’: Original data was the average log2 hybridization signal ratios measured at days 9,11,15 and 20, relative to day 3. The representative CLS value was the area under curve of the average signal curve. Reference [3].
- ‘Gresham-L’ & ‘Gresham-P’: Original data was the half-life of knockout mutant measured for the up and down tag probes. The representative CLS value was the mean half-life of both probes. Reference [4].
- ‘Marek’: Original data was the position-corrected maximum lifespan, which was taken as the representative CLS value. Reference [5].
- ‘Garay’: Original data was the relative lifespan to WT strain, which was taken as the representative CLS value. Reference [6].

- 'Campos-NR' & 'Campos DR': Original data was the relative survival coefficient to WT strain, which was taken as the representative CLS value. When replicates for a single knockout mutation were available, the replicates were averaged. Reference [7].

This process resulted in the nine different datasets compiled in **Table S1**.

REFERENCES

1. Powers RW, Kaeberlein M, Caldwell SD, Kennedy BK, Fields S. Extension of chronological life span in yeast by decreased TOR pathway signaling. Genes Dev. 2006;20:174–84. https://doi.org/10.1101/gad.1381406.

2. Matecic M, Smith DL, Pan X, Maqani N, Bekiranov S, Boeke JD, et al. A microarray-based genetic screen for yeast chronological aging factors. PLoS Genet. 2010;6:e1000921. https://doi.org/10.1371/journal.pgen.1000921.

3. Fabrizio P, Hoon S, Shamalnasab M, Galbani A, Wei M, Giaever G, et al. Genome-wide screen in Saccharomyces cerevisiae identifies vacuolar protein sorting, autophagy, biosynthetic, and tRNA methylation genes involved in life span regulation. PLoS Genet. 2010;6:e1001024. https://doi.org/10.1371/journal.pgen.1001024.

4. Gresham D, Boer VM, Caudy A, Ziv N, Brandt NJ, Storey JD, et al. System-level analysis of genes and functions affecting survival during nutrient starvation in Saccharomyces cerevisiae. Genetics. 2011;187:299–317. https://doi.org/10.1534/genetics.110.120766.

5. Marek A, Korona R. Restricted pleiotropy facilitates mutational erosion of major life-history traits. Evolution (N Y). 2013;67:3077–86. https://doi.org/10.1111/evo.12196.

6. Garay E, Campos SE, González de la Cruz J, Gaspar AP, Jinich A, DeLuna A. High-Resolution Profiling of Stationary-Phase Survival Reveals Yeast Longevity Factors and Their Genetic Interactions. PLoS Genet. 2014;10:e1004168. https://doi.org/10.1371/journal.pgen.1004168.

7. Campos SE, Avelar-Rivas JA, Garay E, Juárez-Reyes A, DeLuna A. Genomewide mechanisms of chronological longevity by dietary restriction in budding yeast. Aging Cell. 2018;17:e12749. https://doi.org/10.1111/acel.12749.
